# The effects of gut microbiome manipulation on glycemic indices in patients with non-alcoholic fatty liver disease: a comprehensive umbrella review

**DOI:** 10.1038/s41387-024-00281-7

**Published:** 2024-05-10

**Authors:** Azin Vakilpour, Ehsan Amini-Salehi, Arman Soltani Moghadam, Mohammad-Hossein Keivanlou, Negin Letafatkar, Arman Habibi, Mohammad Hashemi, Negar Eslami, Reza Zare, Naeim Norouzi, Hamed Delam, Farahnaz Joukar, Fariborz Mansour-Ghanaei, Soheil Hassanipour, Sandeep Samethadka Nayak

**Affiliations:** 1 Guilan University of Medical Sciences, Rasht, Iran; 2 Gastrointestinal and Liver Diseases Research Center, Guilan University of Medical Sciences, Rasht, Iran; 3 Tehran University of Medical Sciences, Tehran, Iran; 4 Student Research Committee, School of Medicine, Guilan University of Medical Sciences, Rasht, Iran; 5 Student Research Committee, Faculty of Medicine, Hormozgan University of Medical Sciences, Bandar Abbas, Iran; 6 Student Research Committee, Larestan University of Medical Sciences, Larestan, Iran; 7 Department of Internal Medicine, Yale New Haven Health Bridgeport Hospital, Bridgeport CT, USA

**Keywords:** Gastrointestinal diseases, Endocrine system and metabolic diseases

## Abstract

**Background:**

Type 2 diabetes mellitus (T2DM) is a significant risk factor for non-alcoholic fatty liver disease (NAFLD). Increased fasting blood sugar (FBS), fasting insulin (FI), and insulin resistance (HOMA-IR) are observed in patients with NAFLD. Gut microbial modulation using prebiotics, probiotics, and synbiotics has shown promise in NAFLD treatment. This meta-umbrella study aimed to investigate the effects of gut microbial modulation on glycemic indices in patients with NAFLD and discuss potential mechanisms of action.

**Methods:**

A systematic search was conducted in PubMed, Web of Science, Scopus, and Cochrane Library until March 2023 for meta-analyses evaluating the effects of probiotics, prebiotics, and synbiotics on patients with NAFLD. Random-effect models, sensitivity analysis, and subgroup analysis were employed.

**Results:**

Gut microbial therapy significantly decreased HOMA-IR (ES: −0.41; 95%CI: −0.52, −0.31; *P* < 0.001) and FI (ES: −0.59; 95%CI: −0.77, −0.41; *P* < 0.001). However, no significant effect was observed on FBS (ES: −0.17; 95%CI: −0.36, 0.02; *P* = 0.082). Subgroup analysis revealed prebiotics had the most potent effect on HOMA-IR, followed by probiotics and synbiotics. For FI, synbiotics had the most substantial effect, followed by prebiotics and probiotics.

**Conclusion:**

Probiotics, prebiotics, and synbiotics administration significantly reduced FI and HOMA-IR, but no significant effect was observed on FBS.

## Introduction

Nonalcoholic fatty liver disease (NAFLD) is known as the leading cause of chronic liver disease worldwide [1]. This condition is characterized by the accumulation of fat in the liver, exceeding 5% of hepatic volume, in individuals without specific factors such as excessive alcohol consumption, hepatotoxic drugs, autoimmune disorders, and viral hepatitis [2]. While NAFLD often manifests without specific symptoms in many individuals, it has the potential to develop into more severe conditions such as nonalcoholic steatohepatitis (NASH), advanced fibrosis, cirrhosis, hepatocellular carcinoma, and, in extreme cases, leading to mortality [1, 3, 4].

The global prevalence of NAFLD has risen steadily, increasing from 25.5% before 2005 to 37.8% after 2016, with an overall reported prevalence of 32.4% [5, 6]. In addition, the total incidence of NAFLD stands at 46.9 cases per 1000 person-years, with rates of 29.6 and 70.8 cases per 1000 person-years in women and men, respectively [6]. The prevalence of NAFLD varies considerably among countries, with Latin America, the Middle East, and North Africa standing out as regions with the highest reported prevalence rates at 44%, 37%, and 36.53%, respectively [7].

NAFLD is closely linked with other metabolic diseases, particularly diabetes mellitus (DM) [8]. A recent meta-analysis showed that individuals with DM have a threefold higher likelihood of developing NAFLD [2]. Furthermore, type 2 diabetes mellitus (T2DM) emerged as one of the most pivotal predictors of adverse clinical outcomes in patients with NAFLD, encompassing conditions such as nonalcoholic steatohepatitis (NASH), liver fibrosis, and mortality [9–12]. The relationship between T2DM and NAFLD appears to be bidirectional, sharing common pathogenic mechanisms [13]. Previous investigations have demonstrated that patients with impaired glycemic indices, such as elevated levels of fasting blood glucose (FBS), fasting insulin (FI), and Homeostasis Model Assessment-Estimated Insulin Resistance (HOMA-IR), face an elevated risk of developing NAFLD [2, 14, 15].

To date, no confirmed agent has been established for NAFLD treatment, and there is a lack of effective medication for its management [16]. Thus, NAFLD management in the clinic mainly relies on lifestyle alterations, and reducing metabolic risk factors [1, 4].

Recently, the impact of gut microbiota on various diseases has been extensively investigated [17, 18]. As interest in the mechanisms of the gut-liver axis and gut microbiota grows, researchers are uncovering that the influence of the gut microbiome extends beyond the gastrointestinal tract. It is now suggested that NAFLD is closely linked with the bacterial population in the small intestine [19]. Both animal and clinical studies have illustrated that gut microbiota can exert its effects on NAFLD through diverse mechanisms. These mechanisms include the modulation of the intestinal barrier, B-cell function in the pancreas, maintenance of bile salt homeostasis, control of appetite, regulation of immune cell functions, reduction of inflammation, and maintenance of glucose homeostasis [20–23].

Several experimental and clinical studies have evaluated the effects of alterations in the gut microbiome population using probiotics (live micro-organisms), prebiotics (indigestible foods), and synbiotics (combination of probiotics and prebiotics) on glycemic parameters and other clinical outcomes of NAFLD. Although several meta-analyses have been conducted in this regard, the results are controversial [24–26].

Due to inconsistent results among the previous meta-analyses, there is a need for an umbrella review to converge the findings of prior meta-analyses. This involves assessing their quality and deriving a conclusive summary based on the latest evidence. In this umbrella review, our objective was to evaluate the impact of gut microbiome-targeted therapy on the glycemic indices of patients with NAFLD by synthesizing information from previous meta-analyses in this specific domain.

## Materials and methods

We conducted the present meta-umbrella study evaluating the effects of gut microbial therapy by administration of prebiotics, probiotics, and synbiotics on the glycemic profile of NALFD individuals according to Preferred Reporting Items for Systematic Reviews and Meta-analysis (PRISMA) guideline [27].

A previous protocol was registered in the International Prospective Register of Systematic Reviews (PROSPERO) (CRD42022346998).

### Search strategy and study selection

We designed the search strategy based on the PICOS (population, intervention, comparators, outcomes, and study design) format, presented in Table 1.

**Table 1:** PICOS criteria of the search strategy.

Population	Patients with non-alcoholic fatty liver disease
Interventions	Prebiotics, probiotics, and synbiotics
Comparators	Placebo or no treatment
Outcome	Fasting blood sugar (FBS) levels, Fasting Insulin (FI) level, homeostasis model assessment-estimated insulin resistance (HOMA-IR)
Study design	Meta-analysis

In the next step, we designed a search formula for four international databases, including PubMed, Scopus, Web of Science, and Cochrane Library, from inception up to 3 March 2023 using keywords including “nonalcoholic fatty liver disease”, “nonalcoholic steatohepatitis”, “prebiotics”, probiotics”, synbiotics”, “gut microbiome”, “gut modulation”, “systematic review”, and “meta-analysis”. The search formula for each database is presented in Table 2. To increase search quality, we consulted two content experts in this regard. To increase the sensitivity of the search, we conducted hand-searching on the reference list of included studies.

**Table 2:** Search strategy for each international database.

PubMed	((“Non-alcoholic Fatty Liver Disease”[Mesh]) AND (((“Prebiotics”[Mesh]) OR (“Probiotics”[Mesh])) OR (“Synbiotics”[Mesh]))) AND ((“Meta-Analysis” [Publication Type]) OR (“Systematic Review” [Publication Type]))
ISI/Web of Science	((“Non alcoholic Fatty Liver Disease” OR “NAFLD” OR “ Nonalcoholic Fatty Liver Disease” OR “Fatty Liver, Nonalcoholic” OR “Fatty Livers, Nonalcoholic” OR “Liver, Nonalcoholic Fatty” OR “Livers, Nonalcoholic Fatty” OR “Nonalcoholic Fatty Liver” OR “Nonalcoholic Fatty Livers” OR “Nonalcoholic Steatohepatitis” OR “Nonalcoholic Steatohepatitides” OR “Steatohepatitides, Nonalcoholic” OR “Steatohepatitis, Nonalcoholic”) AND (“Prebiotics” OR “Probiotics” OR “Synbiotics”) AND (“systematic reviews” OR “meta-analysis”))
Scopus	TITLE-ABS-KEY (“Non alcoholic Fatty Liver Disease” OR “NAFLD” OR “ Nonalcoholic Fatty Liver Disease” OR “Fatty Liver, Nonalcoholic” OR “Fatty Livers, Nonalcoholic” OR “Liver, Nonalcoholic Fatty” OR “Livers, Nonalcoholic Fatty” OR “Nonalcoholic Fatty Liver” OR “Nonalcoholic Fatty Livers” OR “Nonalcoholic Steatohepatitis” OR “Nonalcoholic Steatohepatitides” OR “Steatohepatitides, Nonalcoholic” OR “Steatohepatitis, Nonalcoholic”) AND TITLE ABS-KEY (“Prebiotics” OR “Probiotics” OR “Synbiotics”) AND TITLE-ABS-KEY (“systematic reviews” OR “meta-analysis”)
Cochrane library	#1 ((“Non alcoholic Fatty Liver Disease” OR “NAFLD” OR “ Nonalcoholic Fatty Liver Disease” OR “Fatty Liver, Nonalcoholic” OR “Fatty Livers, Nonalcoholic” OR “Liver, Nonalcoholic Fatty” OR “Livers, Nonalcoholic Fatty” OR “Nonalcoholic Fatty Liver” OR “Nonalcoholic Fatty Livers” OR “Nonalcoholic Steatohepatitis” OR “Nonalcoholic Steatohepatitides” OR “Steatohepatitides, Nonalcoholic” OR “Steatohepatitis, Nonalcoholic”)):ti,ab,kw #2 (“Prebiotics” OR “Probiotics” OR “Synbiotics”) #1 AND #2

### Inclusion and exclusion criteria

Meta-analyses evaluating the effect of prebiotics, probiotics, or synbiotics on patients with NAFLD were eligible to be included in our study. Systematic reviews without meta-analysis, narrative reviews, randomized-control trials, case-control, cohort, and cross-sectional studies were excluded. In addition, letters to the editor, commentaries, and animal studies were also excluded.

### Quality assessment

Two independent reviewers (E-AS and M-HK) evaluated the methodological quality of studies based on the AMSTAR 2 checklist [28]. Any disagreements were finally resolved by the third researcher (SH). This checklist comprises 16 questions regarding different aspects of systematic reviews and meta-analyses. Answers to the questions could be “Yes”, “Partial Yes”, and “No” based on the reviewers’ decisions. The final score is qualitatively reported as “High”, “Moderate”, “Low”, and “Critically low”.

### Data extraction

Two independent members of the team (EA-S and M-HK) first screened and selected the articles regarding the inclusion criteria of the present study. Then they extracted data from included studies and entered the information into a predesigned Excel sheet form.

Name of the first author, year of publication, name of the journal, number of included participants and studies, model of analysis, software of analysis, effect size (ES), and 95% confidential interval (95%CI) regarding glycemic indices, searched databases and date of the search were extracted. Any disagreements in the data extraction process were resolved by the third researcher (SH).

### Data synthesis and statistical analysis

Comprehensive meta-analysis version 3 (CMA 3) was used to analyze the present study. ES and 95%CI regarding each glycemic index from each included study were used to assess the final summary.

The heterogeneity between studies was evaluated using I^2^ statistics and Cochrane’s Q-test. The significant level of heterogeneity was defined as I^2^ > 50% and *P* value < 0.1. We used random-effect model for the final analysis. If a study evaluated at least two interventions (prebiotics, probiotics, or synbiotics), we extracted each intervention data and analyzed it as an independent effect size.

To find the effect of different factors on the results, we performed various subgroup analyses regarding the type of intervention, reporting units (WMD, SMD, and MD), country of study, study quality, funding source, sample size range, and presence of previously registered protocol. We performed sensitivity analysis to assess each study’s effect on the total summary.

To assess publication bias, we assessed the asymmetry of the funnel plots and performed the Eggers’ regression test; *P*-value < 0.1 was considered the level of significance [29, 30]. In addition, we performed trim and fill analysis to see the stability of the results for possible publication bias.

## Results

### Study selection

**Fig. 1:**
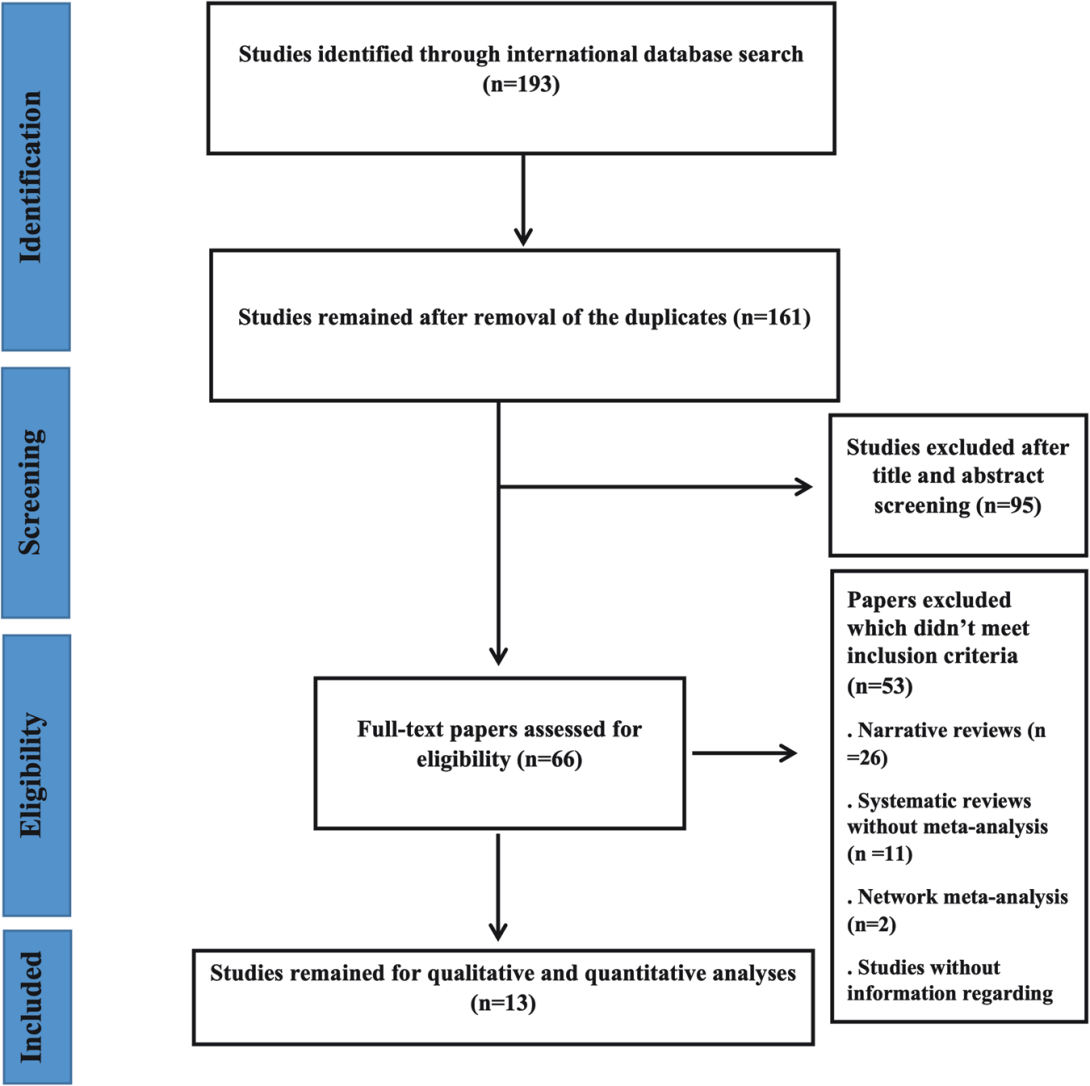
Study selection process. The flowchart diagram.

A total of 193 studies were found after searching the international databases. Thirty-two studies were duplicated and omitted. The remaining 161 studies went for the title and abstract screening, and at this stage, 95 studies were excluded. By evaluating the full text of the remaining articles, a total of 13 studies were finally included in our meta-umbrella study (Fig. 1).

### Characteristics of the studies

The detailed information of the included studies is presented in Table 3. Among the 13 studies that were eligible for our meta-umbrella, six were from China [25, 26, 31–34], three were from the USA [24, 35, 36], and the remaining four studies were from Iran [37], India [38], Poland [39], and France [40]. The number of included studies and total sample size varied between 4–29 and 134–2110, respectively. The treatment duration ranged from 2–48 weeks.

**Table 3:** Characteristics of included studies.

First author/Year of publication	Journal	Country	Previous registered protocol	Funding status	Data bases	Date of search	Number of included studies	Total sample size	Intervention	Duration of treatment	Outcomes	Software of analysis	Model of analysis	Quality of studies
Hadi, 2019 [37]	Critical Reviews in Food Science and Nutrition	Iran	Not reported	No funding	PubMed, Scopus, ISI Web of science and Google Scholar	Up to December, 2017	11	419	Synbiotics	8 to 28 weeks	FBS, HOMA-IR, FI	STATA	Random effect model	Low quality
Huang, 2022 [31]	Computational and Mathematical Methods in Medicine	China	Not reported	Funded	Embase, PubMed, and Web of Science	Up to December, 2021	24	1403	Probiotics	4 to 24 weeks	FBS, HOMA-IR, FI	Revman	Fixed effect model for non-heterogenic and random effect model for heterogenic studies	Low quality
Koutnikova, 2019 [40]	BMJ Open	France	(PROSPERO) CRD42016033273	Funded	PubMed/Medline, EMBASE and the Cochrane Central	1990 to Up to June 2018	12	660	Probiotics	2 to 28 weeks	FBS, HOMA-IR, FI	STATA	Random effects model	High quality
Khan, 2019 [35]	European Journal of Gastroenterology & Hepatology	USA	Not reported	No funding	PubMed/Medline, and Google Scholar	Up to June, 2018	12	782	Probiotics and synbiotics	8 to 24 weeks	FBS	Revman	Random effects model	Critically Low quality
Lavekar, 2017 [38]	Euroasian Journal of Hepato-gastroenterology	India	Not reported	No funding	PubMed, Cochrane, Embase,	Up to February, 2016	7	296	Probiotics	8 to 28 weeks	HOMA-IR	NCSS	Fixed effect model	Critically Low quality
Li, 2022 [32]	Frontiers in Public Health	China	(PROSPERO) CRD42021288543	Funded	PubMed, Embase, the Cochrane Library, Clinical trails.gov, and China National Knowledge Infrastructure	January, 2000 to September, 2021	29	2110	Probiotics, prebiotics, synbiotics	8 to 24 weeks	FBS, HOMA-IR, FI	Revman	Fixed model for non-heterogenic and random model for heterogenic studies	Low quality
Liu, 2019 [33]	Digestive Diseases and Sciences	China	Not reported	Not reported	PubMed, Cochrane, and Embase/	Up to April, 2019	15	782	Probiotics and synbiotics	8 to 28 weeks	FBS, HOMA-IR	Revman	Random effects model	Critically Low quality
Ma, 2013 [36]	World Journal of Gastroenterology	USA	Not reported	Not reported	Medline, Embase, Web of Science, Chinese Biomedicine Database and the China Journal Full Text	Not reported	4	134	Probiotics	8 to 24 weeks	FBS, HOMA-IR	Revman	Fixed model for non-heterogenic and random model for heterogenic studies	Critically Low quality
Sharpton, 2019 [24]	American Journal of Clinical Nutrition	USA	(PROSPERO) CRD42018091455	Funded	PubMed/Medline, Embase, and the Cochrane Library	Up to December, 2018	21	1252	Probiotics and synbiotics	8 to 28 weeks	HOMA-IR	STATA	Random effects model	High quality
Stachowska, 2020 [39]	Nutrients	Poland	Not reported	No funding	PubMed/MEDLINE, Embase, clinicaltrials.gov, Cinahl, Web of Science	Up to March 2020	6	242	Prebiotics	10 and 12 weeks	HOMA-IR, FI	CMA	Random effects model	Low quality
Tang, 2019 [25]	Therapeutic Advances in Gastroenterology	China	(PROSPERO) CRD42019128193	Funded	PubMed, Embase, the Cochrane Library, the Web of Science; China National Knowledge Infrastructure (CNKI), Wan Fang Data, and VIP	Up to April, 2019	22	1356	Probiotics	4 to 24 weeks	FBS, FI	STATA	Fixed model for non-heterogenic and random model for heterogenic studies	High quality
Xiao, 2019 [26]	Gastroenterology Research and Practice	China	Not reported	Funded	PubMed, Embase, Cochrane Library, Web of Science, OVID, China National Knowledge Infrastructure, VIP Database for Chinese Technical Periodicals, China Biology Medicine disc, and Wan fang Database	Up to April, 2019	28	1555	Probiotics	4 to 28 weeks	FBS, HOMA-IR, FI	Revman, STATA	Random effects model	Critically Low quality
Yang, 2021 [34]	Expert Review of Gastroenterology & Hepatology	China	Not reported	No funding	PubMed, Cochrane, Medline, Web of Science and Embase	Up to April, 2021	9	352	Probiotics	8 to 48 weeks	HOMA-IR	Revman	Fixed model for non-heterogenic and random model for heterogenic studies	Critically Low quality

Seven studies evaluated probiotics as their intervention [25, 26, 31, 34, 36, 38, 40], one study assessed synbiotics as intervention [37], one study evaluated prebiotics as intervention [39], and three studies evaluated probiotics and synbiotics as interventions [24, 33, 35]. One study considered probiotics, prebiotics, and synbiotics as interventions [32].

FBS, HOMA-IR, and FI were assessed in 9 [25, 26, 31–33, 35–37, 40], 11 [24, 26, 31–34, 36–40], and 7 [25, 26, 31, 32, 37, 39, 40] studies, respectively. Detailed information of the studies’ characteristics and quality is presented in Table 3.

Based on AMSTAR 2 checklist, three [24, 25, 40] studies had high quality, and four [31, 32, 37, 39], and six [26, 33–36, 38] studies had low and critically low quality, respectively. Detailed information of the studies’ quality is presented in Table S1.

### The effects of microbial therapy on FBS

**Fig. 2:**
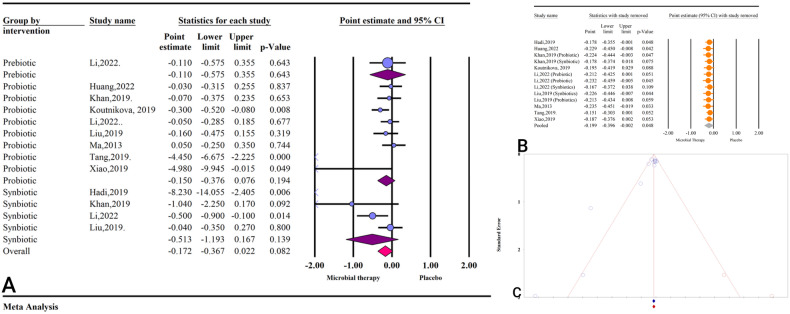
The effects of microbial therapy on FBS. **A** Forest plot for the effect size and 95% confidential interval of gut microbial therapy on serum FBS level in patients with NAFLD. **B** The results of sensitivity analysis. **C** The results of publication bias with two imputed studies (red dots).

The effect of microbial therapy (by administering prebiotics, probiotics, or synbiotics) on FBS was insignificant based on the results of 9 studies with 13 effect sizes (ES: −0.17; 95%CI: −0.36, 0.02; *P* = 0.082) (Fig. 2A). The results were accompanied by significant heterogeneity (I^2^ = 66.70, *P* < 0.001).

The results of sensitivity analysis showed that the effects of microbial therapy on FBS would be significant by removal of Ma [36] (ES: −0.23; 95%CI: −0.45, −0.01; *P* = 0.033), Hadi [37] (ES: −0.17; 95%CI: −0.35, 0.00; *P* = 0.048) or Huang [31] (ES: −0.22; 95%CI: −0.45, 0.00; *P* = 0.042) (Fig. 2B).

The result of subgroup analysis revealed none of the probiotics (ES: −0.15; 95%CI: −0.37, 0.07; *P* = 0.194), prebiotics (ES: −0.11; 95%CI: −0.57, 0.35; *P* = 0.643) and synbiotics (ES: −0.51; 95%CI: −1.19, 0.16; *P* = 0.139) could significantly decrease FBS (Table 4).

**Table 4:** Results of subgroup analysis.

Variable	Subgroups		Number of studies	ES with 95%CI, *P* value	I^2^ (%)	*P-*value of heterogeneity
FBS	Total effect		13	(−0.17; −0.36, 0.02), 0.082	66.70	<0.001
	Intervention type	Probiotics	8	(−0.15; −0.37, 0.07), 0.194	69.45	0.002
		Synbiotics	4	(−0.51; −1.19, 0.16), 0.139	75.21	0.007
		Prebiotics	1	(−0.11; −0.57, 0.35), 0.643	0.00	1.000
	Units of reported	MD	3	(−0.23; −0.75, 0.28), 0.381	78.84	0.009
		SMD	7	(−0.13; −0.27, 0.00), 0.048	6.51	0.378
		WMD	3	(−2.76; −6.58, 1.06), 0.157	89.60	<0.001
	Country	China	9	(−0.16; −0.39, 0.06), 0.161	66.50	0.002
		USA	2	(−0.37; −1.25, 0.50), 0.408	56.91	0.128
		Others	2	(−3.70; −11.40, 3.98), 0.345	85.93	0.008
	Previous registered protocol	Yes	5	(−0.34; −0.70, 0.02), 0.067	78.33	0.001
		No	8	(−0.10; −0.33, 0.12), 0.374	52.71	0.039
	Quality of studies	Critically low	6	(−0.08; −0.28, 0.11), 0.391	30.44	0.207
		Low	5	(−0.18; −0.49, 0.13), 0.255	65.62	0.020
		High	2	(−2.22; −6.27, 1.83), 0.283	92.44	<0.001
	Fund	Yes	7	(−0.28; −0.59, 0.02), 0.068	74.58	0.001
		No	3	(−1.08; −2.81, 0.64), 0.218	79.45	0.008
		Not reported	3	(−0.04; −0.22, 0.13), 0.607	0.00	0.638
	Sample size Range	<300	6	(−0.11; −0.36, 0.14), 0.404	54.41	0.052
		300–600	5	(−0.28; −0.67, 0.10), 0.155	78.58	0.001
		600<	2	(−1.95; −6.34, 2.43), 0.382	70.64	0.065
HOMA-IR	Total effect		15	(−0.41 −0.52, −0.31), <0.001	0.00	0.530
	Intervention type	Probiotics	9	(−0.42; −0.54, −0.30), <0.001	0.00	0.502
		Synbiotics	4	(−0.36; −0.59, −0.12), 0.002	27.50	0.247
		Prebiotics	2	(−0.48; −0.81, −0.14), 0.005	7.82	0.298
	Units of reported	MD	5	(−0.36; −0.53, −0.18), <0.001	0.00	0.462
		SMD	5	(−0.48; −0.63, −0.34), <0.001	0.00	0.640
		WMD	6	(−0.34; −0.56, −0.12), 0.002	18.04	0.297
	Country	China	9	(−0.42; −0.53, −0.32), <0.001	0.00	0.469
		USA	2	(−0.57; −1.33, 0.18), 0.136	0.00	0.373
		Others	4	(−0.33; −0.62, −0.05), 0.021	25.56	0.258
	Previous registered protocol	Yes	6	(−0.47; −0.62, −0.32), <0.001	0.00	0.834
		No	9	(−0.36; −0.51, −0.22), <0.001	19. 74	0.267
	Quality of studies	Critically low	6	(−0.34; −0.49, −0.20), <0.001	2.62	0.400
		Low	6	(−0.46; −0.61, −0.32), <0.001	9.272	0.357
		High	3	(−0.49; −0.92, −0.05), 0.026	0.00	0.648
	Fund	Yes	8	(−0.47; −0.60, −0.34), <0.001	0.00	0.914
		No	4	(−0.16; −0.56, 0.22), 0.406	55.79	0.079
		Not reported	3	(−0.39; −0.56, −0.21), <0.001	0.00	0.759
	Sample size Range	<200	8	(−0.39; −0.54, −0.24), <0.001	16.39	0.301
		200–400	3	(−0.21; −0.67, 0.25), 0.379	11.69	0.322
		400<	4	(−0.46; −0.61, −0.31), <0.001	0.00	0.896
	FI	Total effect	9	(−0.59; −0.77, −0.41), <0.001	0.00	0.717
	Intervention type	Probiotics	5	(−0.57; −0.82, −0.31), <0.001	7.70	0.363
		Prebiotics	2	(−0.61; −0.92, −0.30), <0.001	0.00	0.519
		Synbiotics	2	(−0.65; −1.10, −0.20), 0.005	0.00	0.524
	Units of reported	MD	2	(−0.75; −1.37, −0.13), 0.017	16.32	0.274
		SMD	6	(−0.54; −0.73 to −0.36), <0.001	0.00	0.835
		WMD	1	(−1.32; −2.43, −0.21), 0.020	0.00	1
	Country	China	6	(−0.53; −0.74, −0.33), <0.001	0.00	0.498
		Others	3	(−0.69; −0.99, −0.38), <0.001	0.00	0.846
	Previous registered protocol	Yes	5	(−0.49; −0.69, −0.28), <0.001	0.00	0.963
		No	4	(−0.83; −1.15, −0.50), <0.001	0.00	0.637
	Quality of studies	Critically low	1	(−1.32; −2.43, −0.21), 0.020	0.00	1
		Low	6	(−0.56; −0.75, −0.36), <0.001	0.00	0.610
		High	2	(−0.60; −0.99, −0.20), 0.003	0.00	0.865
	Fund	Yes	7	(−0.53; −0.73, −0.34), <0.001	0.00	0.627
		No	2	(−0.73; −1.09, −0.38), <0.001	0.00	0.764
	Sample size Range	<250	3	(−0.51; −0.72, −0.29), <0.001	0.00	0.526
		250−500	3	(−0.62; −0.98, −0.25), 0.001	0.00	0.792
		500<	3	(−0.83; −1.27, −0.39), <0.001	0.00	0.389

*ES* effect size, *CI* confidential interval, *MD* mean difference, *WMD* weighted mean difference, *SMD* standard mean difference, *FBS* fasting blood sugar, *HOMA-IR* homeostatic model assessment for insulin resistance, *FI* fasting insulin.

The Egger’s regression test showed significant publication bias (*P* = 0.001), and the results of trim and fill analysis showed accepted results with two imputed studies (ES: −0.19; 95%CI: −0.41, 0.02) (Fig. 2C).

### The effects of microbial therapy on HOMA-IR

**Fig. 3:**
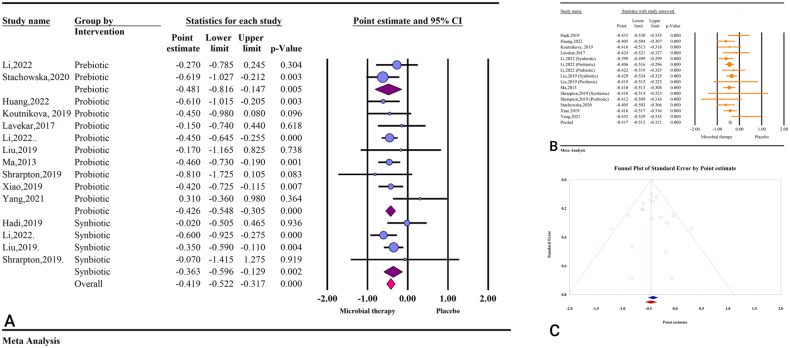
The effects of microbial therapy on HOMA-IR. **A** Forest plot for the effect size and 95% confidential interval of gut microbial therapy on HOMA-IR in patients with NAFLD. **B** The results of sensitivity analysis. **C** The results of publication bias with three imputed studies (red dots).

Based on the result of 11 studies with 15 effect sizes, microbial therapy (by administering prebiotics, probiotics, or synbiotics) could significantly decrease HOMA-IR in patients with NAFLD (ES: −0.41; 95%CI: −0.52, −0.31; *P* < 0.001) (Fig. 3A). The results were homogenous (I^2^ = 00.00%, *P* = 0.530), and sensitivity analysis revealed no significant change after the removal of each study (Fig. 3B).

The results of subgroup analysis revealed that prebiotics (ES: −0.48; 95%CI: −0.81, −0.14; *P* = 0.005) had the most potent effect on HOMA-IR followed by probiotics (ES: −0.42; 95%CI: −0.54, −0.30; *P* < 0.001) and synbiotics (ES: −0.36; 95%CI: −0.59, −0.12; *P* = 0.002) (Table 4). In addition, studies that reported their results in SMD, MD, and WMD showed significant effects of microbial therapy on reducing HOMA-IR (ES: −0.48; 95%CI: −0.63, −0.34; *P* < 0.001, ES: −0.36; 95%CI: −0.53, −0.18; *P* < 0.001, and ES: −0.34; 95%CI: −0.56, −0.12; *P* = 0.002, respectively) (Table 4).

Studies with previously registered protocol showed more substantial effects compared to studies without previously registered protocol (ES: −0.47; 95%CI: −0.62, −0.32; *P* < 0.001 vs. ES: −0.36; 95%CI: −0.51, −0.22; *P* < 0.001) (Table 4). Moreover, studies with high quality reported more potent effects compared to low and critically low quality (ES: −0.49; 95%CI: −0.92, −0.05; *P* = 0.026 vs. ES: −0.46; 95%CI: −0.61, −0.32; *P* < 0.001 vs. ES: −0.34; 95%CI: −0.49, −0.20; *P* < 0.001, respectively) (Table 4).

The results of Egger’s regression test showed no significant publication bias (*P* = 0.228), and the result of trim and fill analysis with three imputed studies was acceptable (ES: −0.41; 95%CI: −0.51, −0.32) (Fig. 3C).

### The effects of microbial therapy on FI

**Fig. 4:**
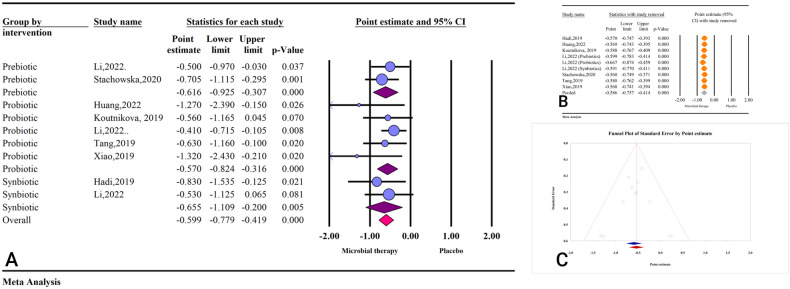
The effects of microbial therapy on FI. **A** Forest plot for the effect size and 95% confidential interval of gut microbial therapy on serum FI level in patients with NAFLD. **B** The results of sensitivity analysis. **C** The results of publication bias with three imputed studies (red dots).

Based on the results of seven studies with nine effect sizes, microbial therapy (by administering prebiotics, probiotics, or synbiotics) could significantly reduce FI in patients with NAFLD (ES: −0.59; 95%CI: −0.77, −0.41; *P* < 0.001) (Fig. 4A). The results were homogenous (I^2^ = 00.00, *P* = 0.717), and the sensitivity analysis result showed no significant change after the removal of each study (Fig. 4B).

The results of subgroup analysis revealed synbiotics (ES: −0.65; 95%CI: −1.10, −0.20; *P* = 0.005) had the most substantial effect on FI, followed by prebiotics (ES: −0.61; 95%CI: −0.92, −0.30; *P* < 0.001) and probiotics (ES: −0.57; 95%CI: −0.82, −0.31; *P* < 0.001) (Table 4). In addition, studies that reported their results in WMD, MD, and SMD showed the significant effect of microbial therapy on NAFLD (ES: −1.32; 95%CI: −2.43, −0.21; *P* = 0.020, ES: −0.54; 95%CI: −0.73, −0.36; *P* < 0.001, ES: −0.75; 95%CI: −1.37, −0.13; *P* = 0.017) (Table 4).

Studies with sample size >500 had the strongest effect, followed by studies with sample size 250−500 and below 250 (ES: −0.83; 95%CI: −1.27, −0.39; *P* < 0.001 vs. ES: −0.62; 95%: −0.98, −0.25; *P* = 0.001 vs. ES: −0.51; 95%CI: −0.72, −0.29; *P* < 0.001) (Table 4). Studies with previously registered protocol had lower effects on FI than those without previously registered protocol (ES: −0.49; 95%CI: −0.69, −0.28; *P* < 0.001 vs. ES: −0.83; 95%CI: −1.15, −0.50; *P* < 0.001) (Table 4). Studies with high quality showed more potent effects compared to studies with low quality (ES: −0.60; 95%CI: −0.99, −0.20; *P* = 0.003 vs. ES: −0.56; 95%CI: −0.75, −0.36; *P* < 0.001) (Table 4).

The results of Egger’s regression test revealed significant publication bias (*P* = 0.003), and the result of trim and fill analysis was acceptable with three imputed studies (ES: −0.53; 95%CI: −0.69, −0.36) (Fig. 4C).

## Discussion

Several studies have reported that NAFLD progression is closely tied to obesity and insulin resistance, and it is expected that the prevalence of NAFLD would rise in synchrony with glucolipid metabolism disorders [41].

Despite recent improvements in our knowledge about NAFLD epidemiology, etiology, and pathogenesis, in the therapeutic field, progress is insignificant. Currently, there is no approved therapy for this disease, and it is necessary to identify appropriate therapeutic targets in parallel to lifestyle modification [42, 43]. Considering the increasing rate of obesity and diabetes in individuals with NAFLD and the bidirectional relationship between NAFLD and T2DM, it is essential to give more attention to this population, especially their glycemic profile [44].

### Findings of our research and other studies

In this meta-umbrella review on 13 studies, we assessed the role of gut microbial therapy as a treatment strategy in individuals with NAFLD concerning their glycemic parameters. Our results showed that gut microbial therapy by administration of prebiotics, probiotics, and synbiotics in patients with NAFLD had beneficial effects on HOMA-IR and FI levels. However, there was no evidence that gut microbial therapy could decrease serum FBS levels in patients with NAFLD. The heterogeneity result for HOMA-IR and FI was insignificant, showing the robustness of the findings in this regard; however, moderate heterogeneity was observed for FBS, which means that our finding for this parameter may be changed by future studies.

We also performed subgroup analysis to assess the effects of prebiotics probiotics and synbiotics separately. Regarding HOMA-IR, prebiotics had the most substantial effect followed by probiotics and synbiotics, whereas synbiotics had the strongest effect on FI followed by prebiotics and probiotics.

This finding shows that various gut microbial therapies have different outcomes on glycemic indices which can be suggestive of different mechanisms of actions and signaling pathways. Further, we did subgroup analyses based on the AMSTAR 2 checklist to assess the impact of the quality of studies. High-quality studies showed more substantial effects on HOMA-IR and FI than low-quality ones. Moreover, studies with sample sizes of more than 400 for HOMA-IR and more than 500 for FI showed more substantial effects. These findings confirm that mixed results in this field could be due to small sample size and poor study quality.

In 70–80% of individuals with NAFLD, elevated blood glucose is a significant finding [45]. Moreover, the population with NAFLD experiences insulin resistance (assessed by HOMA-IR) and elevated levels of FI [46, 47]. In accordance with our study, most studies have proved that gut microbial therapy effectively controlled glycemic indices [3, 48, 49]; however, some results are conflicting [50, 51].

A recent meta-analysis on individuals with NAFLD with 29 randomized controlled trials and 2110 patients showed that gut microbial therapy could remarkably reduce the levels of FBS (*P* = 0.04), HOMA-IR (*P* < 0.001), and IF (*P* = 0.002) [32]. Daubioul et al., in a randomized, double-blind crossover study on seven patients with non-alcoholic steatohepatitis (NASH), found a significant drop in the insulin level four weeks after the intake of oligofructose [52]. Also, another meta-analysis with 24 studies and 1403 participants aimed to investigate the relationship between NAFLD and probiotic supplementation. This study revealed that probiotics supplementation improves HOMA-IR (*P* = 0.03) and reduces FI (*P* = 0.003) [31]. In addition, Gart and colleagues conducted an animal study to assess whether 2-fucosyllactose can reduce NAFLD progress in the presence of obesity. They provided the mice with a high-fat diet for 8 weeks, which made them obese and hyperinsulinemic. They found that 2-fucosyllactose as prebiotic supplementation can significantly reduce FI and HOMA-IR, parallel to diminishing intrahepatic diacylglycerols [53].

A study in Iran enrolled 138 patients with NAFLD aged 18–60 years and randomly divided them into two groups for 16 weeks of treatment duration. One group took sitagliptin 50 mg daily plus a placebo, and another took sitagliptin 50 mg daily plus synbiotics. The result of the study revealed that the combination of sitagliptin with synbiotics compared to sitagliptin alone significantly reduced the FBS levels (*P* < 0.001) [54].

On the other hand, Morvaridzadeh et al. administered vitamin D and probiotics co-supplementation in enriched yogurt among patients with NAFLD. Their results showed no significant effects on glycemic parameters compared to the control group after 12 weeks [55]. Considering this inconsistency, the study reported that they did not assess fecal bacteria loads and characterize the bacterial profile of the intestine before and after the intervention [55]. Moreover, a systematic review and network meta-analysis was conducted on 26 RCTs comprising 1230 adults aged ≥18 years and 159 children aged 6–18 years to compare the effects of synbiotics, probiotics, and prebiotics on FBS and HOMA-IR of patients with NAFLD. Their study indicated that among adults, synbiotics provided the most considerable effects on reducing FBS; however, none of them had a significant impact on HOMA-IR changes [56].

We believe that the different results of such studies can be attributed to the variation of NAFLD stages among individuals, basal microbiota population before the intervention, type of supplements and dosage, basic levels of glycemic parameters, duration of the intervention, and sample size [57].

It is interesting to point out that the promising effect of gut microbial therapy on Glycemic profile was also observed in other populations. A randomized control trial study by Mirmiranpour et al. on individuals with type 2 diabetes revealed that probiotics and synbiotics could significantly reduce blood glucose levels compared with control (*P* < 0.01) following three months of supplementation [58]. Tao et al., in a meta-analysis study of 15 RCTs with 902 patients with diabetes, revealed that probiotics supplements could significantly reduce FBS and HOMA-IR [59]. Similar results were found in patients with polycystic ovarian syndrome (PCOS) [60, 61].

### Possible mechanism of actions

**Fig. 5:**
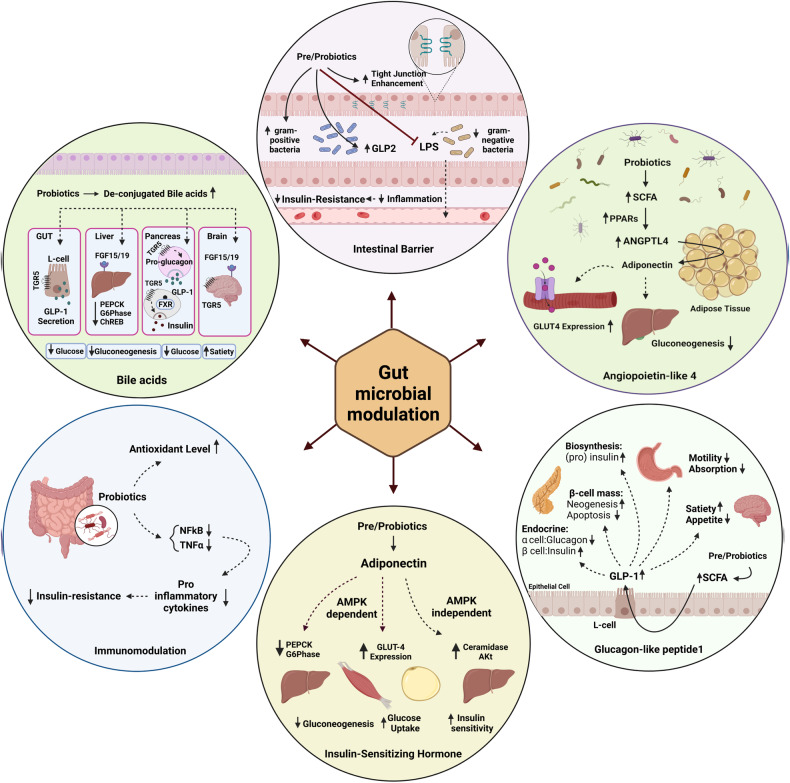
Mechanisms of action. Mechanisms of action on how prebiotic, probiotic, and synbiotic treatment can improve glycemic indices in patients with NAFLD by modulating gut microbiome population.

Although a considerable amount of evidence that has been collected on subjects with NAFLD confirms the effectiveness of gut microbial therapies for glycemic parameters, as mentioned earlier, it is not precisely clear how gut microbial activity contributes to improving glycemic parameters. Here in, we summarize some potential mechanisms that are involved:

Probiotics can promote intestinal tight junctions, decrease intestinal permeability, and reduce inflammation and subsequent insulin resistance [22, 62]. Moreover, probiotics induce adiponectin production, improve insulin sensitivity, and decrease gluconeogenesis via Adenosine monophosphate-activated protein kinase (AMPK) dependent and independent pathways [21, 63]. Probiotics facilitate the synthesis of short-chain fatty acids (SCFAs), including butyrate, acetate, and propionate. SCFAs play a crucial role in glucose hemostasis by increasing the production of gut hormones, including glucagon-like peptide-1 (GLP-1), glucagon-like peptide-2 (GLP-2), and peptide-YY by activating intestinal G-protein-coupled receptors. Also, probiotics affect glycemic profile in patients by activating peroxisome proliferator-activated receptor gamma (PPAR-γ) and angiopoietin-like 4, resulting in improving glucose tolerance and decreasing blood glucose level [64]. Deconjugated bile acids have essential roles in glucose hemostasis, and their synthesis is influenced by gut microbiota [65]. Immunomodulation, regulating the production of inflammatory cytokines, and reducing oxidative stress are other mechanisms of action of probiotics [66]. The mentioned mechanisms will be discussed in detail in the following (Fig. 5).

#### Improving intestinal barrier and inflammation reduction

Elevated LPS production and its translocation from the intestinal lumen to the circulation due to dysbiosis deteriorates insulin signaling by binding to Toll-like receptor 4 (TLR-4), which stimulates transcription of nuclear factor kappa B (NF-kB) and production of pro-inflammatory cytokines [67]. Probiotics improve glucose tolerance, insulin secretion, and inflammation by regulating gut permeability via maintaining tight junctions, changing gram-negative to positive microbiomes ratio, increasing GLP2 secretion, and promoting the health of intestinal cells by providing nutrients [68–71] (Fig. 5).

#### Production of insulin-sensitizing hormone; adiponectin

One of the mechanisms by which probiotic administration improves insulin sensitivity has been attributed to an increase in the production of insulin-sensitizing hormones such as adiponectin [72]. In a study, Lactobacillus rhamnosus GG (LGG) treatment was accompanied by enhanced glucose tolerance, improved insulin sensitivity via adiponectin secretion, and following AMPK activation [73] (Fig. 5).

#### AMPK-dependent actions of adiponectin

AMPK mediates the beneficial effects of adiponectin through the phosphorylating of target proteins in lipids and carbohydrate metabolism [74]. Increased plasma membrane glucose transporter type 4 (GLUT4) expression via AMPK activation stimulates insulin-dependent glucose uptake in skeletal muscle and adipose tissue [75] (Fig. 5). In addition, AMPK activation leads to gluconeogenesis suppression through downregulating gene expression of phospho-enol-pyruvate-carboxykinase (PEPCK) and glucose-6-phosphatase (G6pase) [76]. This AMPK-dependent effect of adiponectin occurs through the suppression of glycogen synthase kinase 3B and the following reduction in cAMP-response element binding protein (CREB) expression [77] (Fig. 5).

#### AMPK-independent actions of adiponectin

There are also AMPK-independent actions of adiponectin enhancing insulin activity and glucose uptake [78]. Among the vast pathways of adiponectin effects on glycemic profile, one has been attributed to an increase in hepatic ceramidase via AdipoR1 and AdipoR2 [79]. High hepatic ceramide levels have been associated with insulin resistance and elevated hepatic glucose output, mainly by preventing the phosphorylation of Akt [80]. So, the adiponectin-dependent elevation of ceramidase has an insulin-sensitizing effect and reduces hepatic glucose output independent from the AMPK signaling pathway [81] (Fig. 5).

#### Glucagon-like peptide-1

SCFAs trigger the secretion of GLP-1 by activating G-protein-coupled receptors (GPR) [82]. In a study by Yadav et al., an increased level of GLP-1 was observed in the human intestinal L-cells treated with butyrate [83]. GLP-1 has a pleiotropic mechanism of action on the glycemic profile. Activation of GLP-1 receptors on pancreatic beta cells increases cyclic adenosine monophosphate (C-AMP) and intracellular calcium levels leading to insulin exocytosis [84]. Also, GLP-1 enhances insulin biosynthesis and beta cell proliferation by activating protein kinase A and following gene expression [85]. In contrast, GLP-1 inhibits counter-regulatory hormones such as glucagon by affecting pancreatic alpha cells [86]. Activation of GLP-1 receptors also inhibits gastric emptying and food ingestion and decreases appetite and food intake [87]. Promoted resistance to apoptosis and enhanced beta cell survival have been attributed to GLP-1 receptor activation [88] (Fig. 5).

#### Role of Bile acids

Probiotics influence the synthesis of deconjugated bile acids, which are essential mediators in glucose hemostasis [64]. In a clinical study, treatment with Lactobacillus reuteri DSM 17938 increased deconjugated bile acids, accompanied by increased insulin sensitivity and glucose metabolism [89]. These effects are mediated through the activation of two main receptors, including the nuclear farnesoid X receptor (FXR) and the membrane-bound G-protein-coupled receptor (TGR5) [90]. The liver activation of FXR increases glycogen synthesis and inhibits gluconeogenesis through negative regulation of carbohydrate response element-binding protein (ChREBP), PEPCK, and G6Pase [91, 92]. Both FXR and TGR5 activation in the pancreas induce insulin production in beta cells [93]. Increased circulating fibroblast growth factor (FGF) 19 following FXR activation stimulates insulin-independent glycolysis in the brain [94]. A study demonstrated that FGF15 expression in mice’s hypothalamus decreases glucagon secretion from pancreatic alpha cells [95]. Also, BA-TGR5 signaling switches alpha cells from glucagon secretory phenotype to GLP-1-producing cells, providing a paracrine effect on pancreatic beta cells to induce insulin secretion [96] (Fig. 5).

#### Angiopoietin-like 4

Angiopoietin-like 4 synthesis is induced by peroxisome proliferator-activated receptor (PPAR) activation, and SCFAs promote its production through PPARs [97]. In a study, Adenovirus-mediated overexpression of Angiopoietin-like factor 4 (ANGPTL4) improved glucose tolerance and decreased blood glucose level [98]. Induced adiponectin secretion from adipocyte tissue mediates ANGPTL4-suppressing effects on hepatic gluconeogenesis and basal glucose output [99]. In addition, adiponectin induces glucose transporter 4 (GLUT4) expression in skeletal muscles improving insulin sensitivity and glucose uptake [100] (Fig. 5).

#### Immunomodulation

Probiotics are associated with enhanced immunity, decreased pro-inflammatory cytokines, and reduced oxidative stress [101]. In a randomized, double blinded, controlled clinical trial, consumption of probiotic-containing yogurt (L. acidophilus La5 and B. lactis Bb12) was accompanied by a reduction in FBS and HbA1C and increased total antioxidant level compared with the control group [102]. In another study, it was demonstrated that butyrate (one of the main SCFA produced by gut microbiota) inhibits TNF-a secretion and NFkB activation via lipoxygenase signaling pathway, preventing pro-inflammatory cytokines expression under NFkB regulation [103]. Butyrate also suppresses T-cell activation through down-regulation of intracellular cell adhesion molecule-1 by suppressing interleukin (IL)1-B and TNF-a expression [104]. Since the over-production of pro-inflammatory cytokines and oxidative stress has been attributed to insulin resistance and type-2 diabetes, probiotics might have a beneficial effect on improving insulin sensitivity by reducing systemic inflammation (Fig. 5).

### Strengths, limitations, and further suggestions

In the present study, we aimed to comprehensively evaluate the effect of gut microbial therapy by evaluating published relevant meta-analyses. We assessed the effects of prebiotics, probiotics, and synbiotics separately to determine which compound is more potent for enhancing glycemic indices. In addition, we performed various subgroup analyses to see the effects of different determinants, including country of study, quality of studies, sample sizes, and funding, on the results. We also assessed possible pathophysiology regarding the act of microbiome in our body. Our study had some limitations. We did not assess the appropriate dosage of treatment. We highly recommend that in future, researchers perform meta-analyses studies to assess whether the action of probiotics, prebiotics, and synbiotics is dose-dependent. In addition, we did not assess the optimum duration of treatment. We also did not assess the different types of supplements, whether it is better to provide powder to patients or pills. Due to insufficient studies, we could not assess the effects of gut microbial therapy on HbA1C. It is strongly encouraged to fill such gaps by conducting randomized control trials and meta-analysis studies.

## Conclusion

In the present study, we found that gut microbial therapy by administration of probiotics, prebiotics, and synbiotics can significantly reduce FI and HOMA-IR, but the effect on FBS level was not significant. In subgroup analysis, prebiotics had the most substantial effect on HOMA-IR, followed by probiotics and synbiotics. Synbiotics had the most potent effect on FI, followed by prebiotics and probiotics. Our findings further revealed the pronounced ameliorative effects of gut microbial treatment on FI and HOMA-IR in high-quality meta-analyses and those with larger sample sizes. Considering the close relationship between DM and NAFLD and the importance of controlling glycemic indices in patients with NAFLD to prevent the disease progression and occurrence of complications, gut microbial treatment by administration of prebiotics, probiotics, and synbiotics can be considered as a therapeutic option; however, more information is needed regarding other glycemic indices like HbA1c. In addition, it is important to delve further into comprehensive details concerning the dosage, optimal treatment duration, and the specific bacterial species incorporated in probiotics and synbiotics for future studies. A more detailed exploration of these aspects will not only contribute to a deeper understanding of their therapeutic potential but will also provide more precise and effective applications for of healthcare profession.

### Supplementary information

Supplementary

## Data Availability

The datasets used and/or analyzed during the current study are available from the corresponding author on reasonable request.
