# Correction: The effects of gut microbiome manipulation on glycemic indices in patients with non-alcoholic fatty liver disease: a comprehensive umbrella review

**DOI:** 10.1038/s41387-024-00327-w

**Published:** 2024-08-20

**Authors:** Azin Vakilpour, Ehsan Amini-Salehi, Arman Soltani Moghadam, Mohammad-Hossein Keivanlou, Negin Letafatkar, Arman Habibi, Mohammad Hashemi, Narges Eslami, Reza Zare, Naeim Norouzi, Hamed Delam, Farahnaz Joukar, Fariborz Mansour-Ghanaei, Soheil Hassanipour, Sandeep Samethadka Nayak

**Affiliations:** 1 Guilan University of Medical Sciences, Rasht, Iran; 2 Gastrointestinal and Liver Diseases Research Center, Guilan University of Medical Sciences, Rasht, Iran; 3 Tehran University of Medical Sciences, Tehran, Iran; 4 Student Research Committee, School of Medicine, Guilan University of Medical Sciences, Rasht, Iran; 5 Student Research Committee, Faculty of Medicine, Hormozgan University of Medical Sciences, Bandar Abbas, Iran; 6 Student Research Committee, Shiraz University of Medical Sciences, Shiraz, Iran; 7 Department of Internal Medicine, Yale New Haven Health Bridgeport Hospital, Bridgeport CT, USA

**Keywords:** Gastrointestinal diseases, Endocrine system and metabolic diseases

Correction to: *Nutrition and Diabetes* 10.1038/s41387-024-00281-7, published online 10 May 2024

In this article the affiliation details for author Hamed Delam were incorrectly given as Student Research Committee, Larestan University of Medical Sciences, Larestan, Iran but should have been Student Research Committee, Shiraz University of Medical Sciences, Shiraz, Iran.

